# Hydrogen sulphide improves adaptation of *Zea mays* seedlings to iron deficiency

**DOI:** 10.1093/jxb/erv368

**Published:** 2015-07-23

**Authors:** Juan Chen, Fei-Hua Wu, Yu-Ting Shang, Wen-Hua Wang, Wen-Jun Hu, Martin Simon, Xiang Liu, Zhou-Ping Shangguan, Hai-Lei Zheng

**Affiliations:** ^1^State Key Laboratory of Soil Erosion and Dryland Farming on the Loess Plateau, Northwest A&F University, Yangling, Shaanxi 712100, P.R. China.; ^2^Key Laboratory of the Ministry of Education for Coastal and Wetland Ecosystem, College of the Environment and Ecology, Xiamen University, Xiamen, Fujian 361005, P.R. China.; ^3^College of Life and Environmental Sciences, Hangzhou Normal University, Hangzhou, Zhejiang 310036, P.R.China.

**Keywords:** Chlorophyll, chloroplast ultrastructure, hydrogen sulphide (H_2_S), iron-deficient, photosynthesis, phytosiderophore, *Zea mays*.

## Abstract

Hydrogen sulphide (H_2_S) is emerging as a potential molecule involved in physiological regulation in plants. However, whether H_2_S regulates iron-shortage responses in plants is largely unknown. Here, the role of H_2_S in modulating iron availability in maize (*Zea mays* L. cv Canner) seedlings grown in iron-deficient culture solution is reported. The main results are as follows: Firstly, NaHS, a donor of H_2_S, completely prevented leaf interveinal chlorosis in maize seedlings grown in iron-deficient culture solution. Secondly, electron micrographs of mesophyll cells from iron-deficient maize seedlings revealed plastids with few photosynthetic lamellae and rudimentary grana. On the contrary, mesophyll chloroplasts appeared completely developed in H_2_S-treated maize seedlings. Thirdly, H_2_S treatment increased iron accumulation in maize seedlings by changing the expression levels of iron homeostasis- and sulphur metabolism-related genes. Fourthly, phytosiderophore (PS) accumulation and secretion were enhanced by H_2_S treatment in seedlings grown in iron-deficient solution. Indeed, the gene expression of ferric-phytosiderophore transporter (*ZmYS1*) was specifically induced by iron deficiency in maize leaves and roots, whereas their abundance was decreased by NaHS treatment. Lastly, H_2_S significantly enhanced photosynthesis through promoting the protein expression of ribulose-1,5-bisphosphate carboxylase large subunit (RuBISCO LSU) and phosphoenolpyruvate carboxylase (PEPC) and the expression of genes encoding RuBISCO large subunit (*RBCL*), small subunit (*RBCS*), D1 protein (*psbA*), and *PEPC* in maize seedlings grown in iron-deficient solution. These results indicate that H_2_S is closely related to iron uptake, transport, and accumulation, and consequently increases chlorophyll biosynthesis, chloroplast development, and photosynthesis in plants.

## Highlight

H_2_S could help plants coping with iron deficiency through increasing phytosiderophore accumulation and secretion, regulating expression of genes related to iron homeostasis and sulphur metabolism, and consequently enhancing the photosynthesis of maize seedlings.

## Introduction

Iron is an essential nutrient for various cellular and physiological processes in plants. It functions as a component of many important enzymes and proteins that are involved in fundamental biochemical processes, such as respiration, photosynthesis, oxygen transport, and so on (Graziano and Lamattina, 2007). However, iron is often unavailable and difficult for crops to assimilate due to the low solubility of its oxidized form. Therefore, iron deficiency often occurs in plants and leads to interveinal chlorosis, impairment of chlorophyll biosynthesis and chloroplast development, and further affects the photosynthetic apparatus establishment (Ramirez *et al.*, 2011). Thereby, iron availability is directly correlated with plant productivity.

Iron abundance in plant cells is tightly regulated by iron uptake, translocation, and recycling. Under iron-deficient conditions, plants have evolved two separate strategies for iron acquisition. In strategy I, plants respond to iron deprivation by at least three steps including acidification of the rhizosphere by an H^+^-ATPase, reduction of Fe(III) to Fe(II) by ferric-chelate reductase, and uptake of Fe(II) by iron transporters. In contrast, all gramineous plants such as barley and wheat have a distinct strategy (strategy II) for iron acquisition, which is different from that of non-grass species (Curie and Briat, 2003; Walker and Connolly, 2008; Morrissey and Guerinot, 2009). In strategy II, iron acquisition includes (i) biosynthesis of phytosiderophores (mugineic acids, MAs) inside the roots; (ii) secretion of phytosiderophores (PSs) to the rhizosphere; (iii) solubilization of insoluble iron in soils by chelation of PSs; and (iv) uptake of the ferric-phytosiderophore complex by the roots (Ma, 2005; Ueno *et al.*, 2009). However, strategies I and II are not sufficient to support the iron requirement for plant development when iron availability is under a threshold level, thus stress symptoms become evident in iron-deficient plants.

Gramineous plants secrete Fe chelators called mugineic acids family PSs from their roots via transporter of MAs (TOM1) to solubilize Fe in the rhizosphere. Gramineous plants then take up Fe as Fe(III)-MA complexes from the rhizosphere through the action of yellow stripe1 (YS1) transporter at the plasma membrane (Curie *et al.*, 2001). The biosynthetic pathway for MAs in gramineous plants has been elucidated (Mori, 1999). Methionine, which is a precursor of MAs, is converted to 2′-deoxymugineic acid (DMA) via a series of reactions (Mori, 1999). There are many genes involved in these reactions including *NAS1*, *NAS2*, *DMAS1* etc. (Higuchi *et al.*, 1999). TOM1, which is a major facilitator superfamily (MFS) antiporter, was identified as an efflux transporter of DMA in rice (Nozoye *et al.*, 2011). *TOM1* expression is strongly induced in Fe-deficient roots (Nozoye *et al.*, 2011). Similarly, *YS1* expression levels increase in both shoots and roots in Fe-deficient *Zea mays* plants (Ueno *et al.*, 2009). Expression of the ferric-chelate reductase genes *AtFRO2*, *LeFRO1*, and *PsFRO1* is induced by iron starvation in *Arabidopsis*, tomato, and pea, respectively (Robinson *et al.*, 1999; Connolly *et al.*, 2003; Li *et al.*, 2004; Wu *et al.*, 2005). Besides, *IRT1* encoding an iron-regulated transporter—a high-affinity iron uptake system in roots—was found to be the major route for iron entering the cell, and its expression obviously increased in low versus sufficient iron supply (Connolly *et al.*, 2002; Vert *et al.*, 2002). Moreover, iron binding protein (IBP) is ubiquitous in nature because iron transport and delivery to cells for utilization and storage are essential functions in all organisms. Moreover, IBP is also vital in sequestering iron where an over-abundance can quickly lead to oxidative stresses (Strickler-Dinglasan *et al.*, 2006).

Recently, many studies have revealed that H_2_S can act as a signalling molecule similar to nitric oxide (NO) and carbon monoxide (CO) in animals at lower concentrations and can participate in various biological processes (Wang, 2002; Yang *et al.*, 2008). Interestingly, some studies have focused on the physiological function of H_2_S in plants. For instance, it was reported that H_2_S promoted wheat seed germination, alleviated oxidative damage against copper stress, counteracted chlorophyll loss, and alleviated oxidative damage due to osmotic stress in sweet potato seedling leaves (Zhang *et al.*, 2008; Zhang *et al.*, 2009). Furthermore, boron toxicity, salinity toxicity, and aluminium toxicity were alleviated by H_2_S (Wang *et al.*, 2010; Zhang *et al.*, 2010; Wang *et al.*, 2012; Chen *et al.*, 2013). In addition, a low concentration of H_2_S can promote growth of the embryonic root of *Pisum sativum* and act as a regulator of flower senescence (Li *et al.*, 2010; Zhang *et al.*, 2011). Besides, H_2_S induces stomatal closure and participates in the abscisic acid (ABA)-dependent signalling pathway by regulating ATP-binding cassette (ABC) transporter in guard cells (García-Mata and Lamattina, 2010). A previous study also showed that H_2_S enhances photosynthesis through promoting chloroplast biogenesis, photosynthetic enzyme expression, and thiol redox modification in *Spinacia oleracea* seedlings (Chen *et al.*, 2011).

It is known that, H_2_S is endogenously generated during the metabolism of l-cysteine by the enzymes cystathionine β-synthase and cystathionine γ-lyase in plants (Hughes *et al.*, 2009). Indeed, H_2_S is thought to be released from cysteine via a reversible *O*-acetyl-l-serine(thiol)lyase (OAS-TL) reaction in plants (Riemenschneider *et al.*, 2005*a*; Birke *et al.*, 2015). Moreover, the uptake of H_2_S is largely dependent on its rate of metabolism into cysteine by OAS-TL and subsequent assimilation into other organic sulphur compounds (De Kok *et al.*, 1998, 2002; Durenkamp *et al.*, 2007). Therefore, H_2_S is an important compound involved in plant sulphur metabolism. It is noteworthy that S supply could help plants cope with the Fe shortage (Astolfi *et al.*, 2004, 2010, 2012; Zuchi *et al.*, 2012). For instance, Astolfi *et al.* (2010) showed that barley exhibited a positive correlation between the S nutritional status of the plant and its capability of coping with Fe deficiency. Moreover, one of the first responses to Fe deficiency in strategy II plants is the extrusion of PSs in the root rhizosphere in order to chelate and solubilize Fe^3+^ (Astolfi *et al.*, 2010, 2012). PSs are derived from nicotianamine that is synthesized from three molecules of *S*-adenosyl-methionine, thus representing another possible junction between Fe and S metabolism. Under sulphur-deficiency conditions, the release of PSs is reduced (Astolfi *et al.*, 2006*a*, 2010, 2012); however, the question is,whether H_2_S as a sulphur compound or a signalling molecule plays a key role in response to Fe deficiency in plants.

Although iron uptake and translocation are controlled by many components which have been recently characterized at the molecular level (Schmidt, 1999), it is not known how signalling molecules are involved in the response of plants to iron deficiency. Recent studies have shown that NO, as a bioactive free radical molecule, regulates iron metabolism in plants (Graziano *et al.*, 2002; Murgia *et al.*, 2002). Likewise, some evidence has been provided that CO, as an endogenous gaseous molecule, may play an important role in improving plant adaptation to iron deficiency (Kong *et al.*, 2010). However, it is still not clear whether a low concentration of H_2_S, similar to that of NO and CO, is involved in the regulation of iron assimilation and availability in plants.

In this study, compelling data are presented that reveal a novel effect of H_2_S in plant biology, more specifically on iron nutrition. The results support the idea that H_2_S is closely related to iron uptake, transport, and accumulation, and consequently increases chlorophyll biosynthesis, chloroplast development, and photosynthesis in plants.

## Materials and methods

### Plant growth and treatments

Seeds of maize (*Zea mays* L. cv Canner) were first sterilized in 75% ethanol for 3min and then in 10% sodium hypochlorite solution for an additional 10min followed by washing with distilled water and germinating in a soil:vermiculite (1:1) mixture for 5 d. Five-day-old seedlings were transferred to plastic pots (two seedlings per pot) filled with 0.75 l of nutrient solution. The nutrient solution had the following composition: 5.25mM KNO_3_, 7.75mM Ca(NO_3_)_2_, 4.06mM MgSO_4_, and 1.0mM KH_2_PO_4_; micronutrients: 46 μM H_3_PO_4_, 9.18 μM MnSO_4_, 5.4 μM ZnSO_4_, 0.3 μM CuSO_4_, and 2.0 μM Na_2_MoO_4_. Iron was supplied as 50 μM Fe(III)-EDTA or in different concentrations ranging from 10 to 250 μM. Plants were grown in a controlled growth chamber with a light/dark regime of 15/9h, relative humidity of 80%, temperature of 21/27 °C and a photosynthetically active radiation (PAR) of 190 μmol m^−2^ s^−1^.

NaHS was used as exogenous H_2_S donor as described by Hosoki *et al.* (1997). Seedlings were divided into four groups for further treatment. In the first group, 5-d-old seedlings were pre-treated with various concentrations of NaHS (0, 10, 100, 500, 1000 μM) for 8 d and then were grown in nutrient solution containing 50 μM Fe(III)-EDTA for 12 d. In the second group, in order to distinguish the possible roles of H_2_S, HS^-^, Na^+^, or other sulphur-containing components in iron acquirement, a series of chemicals—Na_2_S, Na_2_SO_4_, Na_2_SO_3_, NaHSO_4_, NaHSO_3_, NaAC, cysteine (Cys), glutathione (GSH), and methionine (Met) were used as the controls of NaHS. In the third group, 5-d-old seedlings were pre-treated with the optimal concentration of NaHS for 8 d and then were grown in nutrient solution containing 0, 25, 50, 75, 100, 250 μM Fe(III)-EDTA for 12 d. In the fourth group, seedlings were pre-treated with the optimal concentration of NaHS for 8 d and then were grown in nutrient solution with 1 μM Fe(III)-EDTA (–Fe) and with 50 μM Fe(III)-EDTA (+Fe) for 12 d. All above of solutions were changed every 4 d.

### Chlorophyll quantification

Chlorophyll content was measured according to Lichtenthaler (1987). Maize leaves (0.2g of fresh weight) were powdered with liquid nitrogen, and pigments were extracted using four volumes of 80% (v/v) aqueous acetone until complete bleaching; the content of total chlorophyll was then calculated from the absorbance of leaf chlorophyll extracts at 470, 646, and 663nm.

### Iron quantification

The samples (leaves, stems, and roots) were washed three times with distilled deionized water and then dried at 70 °C for 48h. The samples with weight ranging from 50 to 100mg were placed into the digestion vessels, mixed with 5ml of concentrated HNO_3_ (65–68%), and digested in microwave digestion system (CEM Inc., Mars-V). The solution was finally diluted to a certain volume with distilled deionized water. The content of Fe was analysed by inductively coupled plasma mass spectrometry (ICP-MS, PerkinElmer Inc., Elan DRC-e) (Chen L. *et al.*, 2010).

### Determination of endogenous H_2_S, GSH, and non-protein thiols content

Endogenous H_2_S was measured by the formation of methylene blue from dimethy-p-phenylenediamine in H_2_SO_4_ according to the method described by Sekiya *et al.* (1982) and Chen *et al.* (2011) with some modifications. Reduced GSH was estimated using a kit of GSH reagent (Jiancheng Bioengineering Institute, Nanjing, China) according to the method described by Chen *et al.* (2013). The total content of non-protein thiols (NPTs) in maize seedlings was measured according the Del Longo *et al.* (1993) with minor modifications.

### Measurement of PSs content in root tips

PSs content in root tips was determined according to the method of Reichman and Parker (2007) and Zuchi *et al.* (2012). Maize seedlings were removed from nutrient solution and root tips (about 10mm) were washed, collected, and then homogenized to a fine powder with liquid nitrogen. Distilled water (100 °C) was added to aliquots of the powdered tissue and homogenates were incubated for 10min at 80 °C. Insoluble material was removed by 10min centrifugation in a microliter centrifuge at 12000rpm and the pellet was then re-extracted with boiling water as described above. After a further centrifugation, the supernatant was used for determination of PSs content in root tips using the Fe-binding assay revised by Reichman and Parker (2007).

### Collection of root exudates and determination of PSs release

PSs release from maize seedling roots was analysed by determining PSs content in root washing. Maize seedlings were removed from the nutrient solution and the roots were washed two times for 1min in deionized water. Root systems were submerged into 200ml deionized water for 6h with continuous aeration. PSs content in root washing was determined using the Fe-binding assay revised by Reichman and Parker (2007).

### Transmission electron microscopy

Leaves were cut into 0.5×1mm pieces and immediately fixed with 2.5% glutaraldehyde (in 0.1M sodium phosphate buffer, pH 7.0) at room temperature for 4h. After three 20min rinses, the samples were post-fixed with 1% OsO_4_ in the same buffer for another 4h, followed by three buffer rinses. Samples were dehydrated in an acetone series, embedded in Spurr’s resin, and sectioned with a Leica EM UC6 ultramicrotome (Leica Microsystems GmbH, Wetzlar, Germany). The ultrathin sections (70–90nm) were stained with uranyl acetate and lead citrate. A Philips CM 100 transmission electron microscopy (TEM) (Philips, Eindhoven, Netherlands) at 80kV was used for ultrastructure imaging of chloroplast. At least three seedlings and more than 30 individual chloroplasts were observed for each treatments by the method of Chang *et al.* (2008).

### Leaf gas exchange measurements

Light-response and CO_2_-response curves of photosynthesis (Pn) were measured using a portable photosynthesis system (Li-6400, Li-Cor, Lincoln, NE, USA) on the fourth fully developed leaf of the seedlings. All measurements were conducted in the morning (9:00–11:30) to avoid high temperatures and the air vapour pressure deficit in the afternoon. Light was supplemented using a LED light system. The measurement was carried out on at least six leaves for all treatments in 380μl l^−1^ CO_2_ at room temperature (25°C). The apparent dark respiration (*R*d), light compensation point (*L*cp), light saturation point (*L*sp), apparent quantum yield (*AQE*), and maximal net photosynthetic rate (*P*max) were calculated by modelling the response of leaf Pn to PAR by a non-rectangular hyperbola, as described by Prioul and Chartier (1977).

$$Pn=\frac{AQE\times PAR+P\max-\sqrt{\begin{array}{l} (AQE\times PAR+P\max{)}^{2}\\ -\;4AQE\times \theta \times PAR\times P\max \end{array}}}{2\theta }-Rd$$

where θ is the convexity. The Pn was modelled as a function of intercellular CO_2_ concentration (Ci). This application fits a model curve described by the rectangular hyperbola (Olsson and Leverenz, 1994):

Pn=CE×Ci×PmaxCE×Ci+Pmax−Re,

where Pn is the assimilation rate, *CE* is carboxylation efficiency, Ci is intercellular CO_2_ concentration, *P*max is the assimilation at saturating CO_2_ and *R*e is the respiratory processes (dark and light). Experimental data are fitted by first obtaining initial estimates of *CE* and *R*e values using linear regression over the lower part of the curve and estimating *P*max from the largest value. Subsequently a least-squares fit was obtained and values for *CE*, *R*e, and *P*max were presented.

### SDS-PAGE and western blot analysis

Leaf samples (0.5g) were ground in liquid N_2_ with a mortar and pestle. Total protein was extracted with a buffer containing 50mM phosphate buffer solution (pH7.5), 2% β-mercaptoethanol, 100mM EDTA, 1% PVPP (w/v), and 1% TritonX-100 (v/v). After 15min centrifugation (4 °C, 15000 *g*), the upper phase was transferred to a new centrifuge tube. Two volumes of Tris-saturated phenol (pH 8.0) were added and then the mixture was further vortexed for 30min. Proteins were precipitated by adding five volumes of ammonium sulphate saturated-methanol, and incubated at −20 °C for at least 4h. After centrifugation as described above, the protein pellets were re-suspended and rinsed with ice-cold methanol followed by washing with ice-cold acetone twice, and spun down at 15000 *g* for 10min at 4 °C after each washing. Finally the washed pellets were air-dried and recovered in the lysis buffer containing 62.5mM Tris-HCl (pH 6.8), 2% SDS (v/v), 10% glycerol (v/v), and 2% β-mercaptoethanol (v/v). Protein concentrations were quantified using the Bradford assay (Bradford, 1976).

For western blot analysis, proteins (20 μg from each sample) were separated by SDS-PAGE using 12% (w/v) acrylamide gels according to Laemmli (1970) and electrophoretically transferred to polyvinylidene difluoride (PVDF) membrane for 50min. The membrane was blocked overnight with western blocking buffer (TIANGEN, China). The protein blot was probed with a primary antibody of ribulose-1,5-bisphoshate carboxylase (RuBISCO) large subunit (RuBISCO LSU) (AS03 037-200, Agrisera, Sweden) and phosphoenolpyruvate carboxylase (PEPC) (AS09 458, Agrisera, Sweden) at dilutions of 1:5000 and 1:1000, respectively for 4h at room temperature with agitation. Then the blot was washed three times in PBS with Tween-20 (PBST) solution containing 50mM Tris-HCl (pH 8.0), 150mM NaCl, 0.05% Tween-20 (v/v), followed by incubation with the secondary antibody (anti-rabbit IgG horse radish peroxidase conjugated, Abcam, UK; 1:5000 dilution) for 2h at room temperature. β-actin (1:5000; Santa Cruz, CA, USA) was used as an internal control. The blots were finally washed as above and developed with SuperSigmal West Pico Chemiluminescent Substrate (Pierce, USA) according to the manufacturer’s instructions. Images of the blots were obtained using a CCD imager (FluorSMax, Bio-Rad, USA). The Quantity One software (Bio-Rad, Hercules, CA, USA) was used to determine the optical density value. The comparative optical density value was used to determine the relative amount of protein expression, with the expression of β-actin used as an internal control.

### Total RNA extraction, reverse transcription and quantitative real-time PCR (qRT-PCR)

Leaves and roots (0.5g) were frozen and ground in liquid nitrogen with 2% polyvinylpyrrolidone and extracted with 0.5ml of RNA purification reagent (Invitrogen Inc., CA, USA) by following the manufacturer’s procedure. The RNA concentration was determined by using anultraviolet spectrophotometer (Cary 50, Varian, USA) and RNA integrity was detected by 1% agarose gel electrophoresis. Total RNAs were reverse-transcribed into first-strand cDNAs with M-MLV reverse transcriptase (TaKaRa, Dalian, China). A 10-μl real-time PCR reaction contained the following: 0.6 μl of forward and reverse primers including iron homeostasis-related genes, sulphur metabolism-related genes, and photosynthesis-related genes (Supplementary Table S1, available at *JXB* online), 1 μl of cDNA (equivalent to 10ng of mRNA), and 5 μl of Faststart Universal SYBR Green Master (ROX, Mannheim, Germany). Amplification and detection of dsDNA synthesis of these genes were performed respectively using the PCR conditions as described in Supplementary Table S2, available at *JXB* online. Three independent replicates were performed for each sample. The comparative threshold cycle (C_t_) method was used to determine the relative amount of gene expression. Actin2 was used as an internal control. The mRNA transcriptional abundance value of genes was expressed as 2^−ΔΔCt^ (Livak and Schmittgen, 2001).The LightCycler 480II Real-Time PCR system (Roche, Bern, Switzerland) was used to run qRT-PCR.

### Statistical analysis

For gas exchange measurements, at least six leaves were used. For physiological and biochemical analyses, at least three replicates were included. Statistical significance was tested by one-way or two-way ANOVA with SPSS 13.0 (SPSS Inc., Chicago, IL, UA), and results are expressed as the mean values ± standard error. Post-hoc comparisons were tested using the Tukey test at a significance level of *P*<0.05.

## Results

### H_2_S rather than other sulphur-containing derivatives significantly inhibits the chlorophyll loss in iron-deficient maize plants

**Fig. 1.**
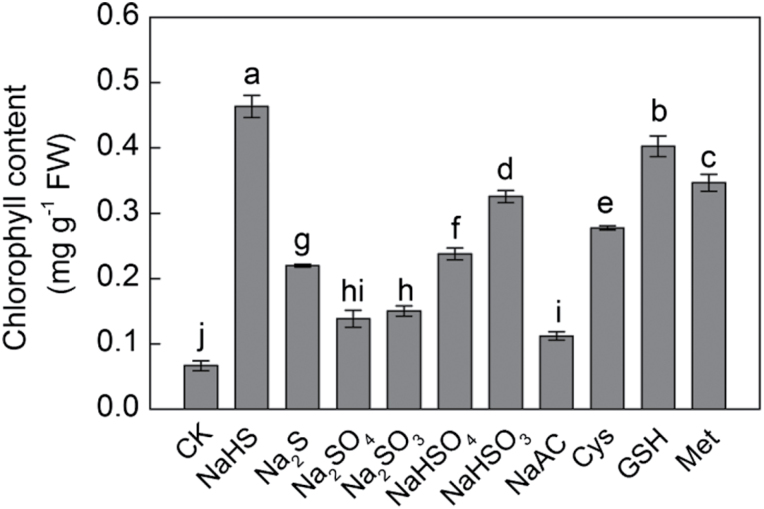
H_2_S but not other sulphur- or sodium-containing compounds derived from NaHS contribute to increased chlorophyll content in iron-deficient maize plants. Maize seedlings were pre-treated with different sulphur-containing compounds for 8 d and then grown in a nutrient solution containing 1 µM Fe(III)-EDTA (–Fe) for 12 d. Data are presented as means ± SE. Columns labelled with different letters indicate significant differences at *P*<0.05.

The first step in studying the role of H_2_S in regulating plant iron assimilation, was to examine its effect on chlorophyll content in maize seedling leaves. In order to distinguish the role of H_2_S from that of other sulphur-containing derivatives and sodium, a series of sulphur- and sodium-containing chemicals including NaHS, Na_2_S, Na_2_SO_4_, Na_2_SO_3_, NaHSO_4_, NaHSO_3_, NaAC, Cys, GSH, and Met were used to treat maize seedlings under –Fe conditions. After treatment for 20 d, Na^+^ or other sulphur-containing compounds, which were used as controls of NaHS, did not cause as great an increase in chlorophyll content as NaHS (Fig. 1). It was also observed that the leaf chlorophyll content of seedlings treated with 100 μM NaHS was much higher than that of other treatments. In addition, the chlorophyll content under GSH treatment increased, but not by as much as the NaHS-induced increases. These results showed that H_2_S rather than other sulphur-containing compounds or sodium was responsible for the increase in chlorophyll content in iron-deficient maize plants. Therefore, NaHS was used as a donor of H_2_S in subsequent experiments.

### H_2_S induces greening and seedling growth in iron-deficient maize plants

**Fig. 2.**
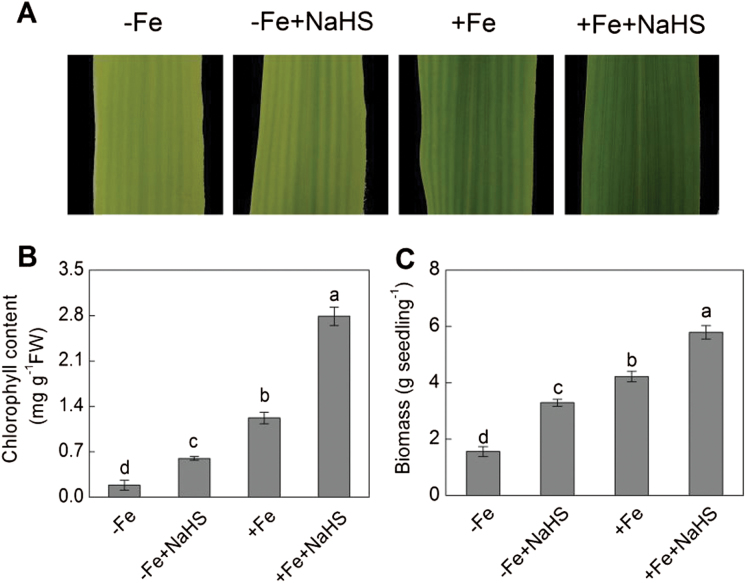
Effect of NaHS on phenotype (A), chlorophyll content (B), and biomass (C) of iron-deficient maize plants. Maize seedlings were pre-treated with 100 µM NaHS for 8 d and then grown in a nutrient solution containing 1 µM Fe(III)-EDTA or 50 µM Fe(III)-EDTA for 12 d. Data are presented as means ± SE. Columns labelled with different letters indicate significant differences at *P*<0.05. –Fe, 1 µM Fe; –Fe+NaHS, seedlings were pre-treated with 100 µM NaHS and then treated with 1 µM Fe; +Fe, 50 µM Fe; +Fe+NaHS, seedlings were pre-treated with 100 µM NaHS and then treated with 50 µM Fe. A colour version of this figure is available at *JXB* online.

**Fig. 3.**
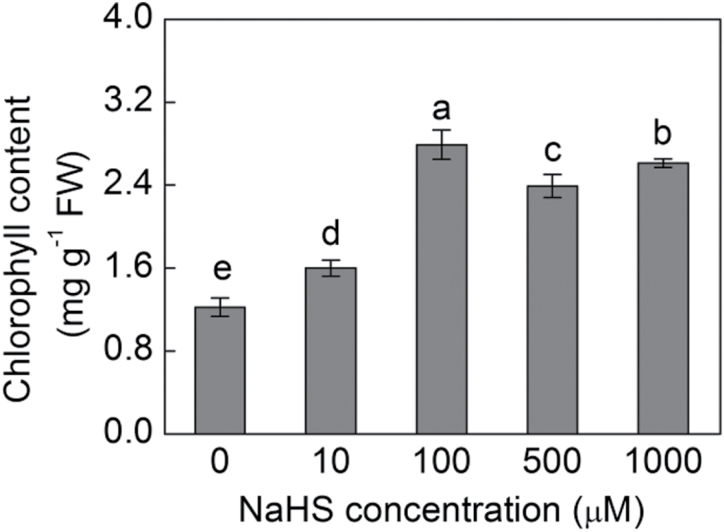
Effect of various NaHS concentrations on chlorophyll content in a dose-dependent manner in iron-deficient maize plants. Maize seedlings were pre-treated with 0, 10, 100, 500, and 1000 µM NaHS for 8 d and then grown hydroponically in a nutrient solution containing 50 µM Fe(III)-EDTA for 12 d. Chlorophyll content in the fourth leaves was measured. Data are presented as means ± SE. Columns labelled with different letters indicate significant differences at *P*<0.05.

Interveinal yellowing or chlorosis of the leaves is a distinguishable symptom associated with iron deficiency. Therefore, it was investigated whether exogenous H_2_S could improve leaf greening in maize plants growing under –Fe or +Fe conditions. Maize plants grown under –Fe conditions showed significant yellowing in the young leaves; however, under +Fe conditions, the young leaves became more healthy to some extent. Importantly, the yellowing leaves could be largely reversed by the addition of 100 μM NaHS, a H_2_S donor (Fig. 2A). The H_2_S-induced leaf greening was estimated by measuring chlorophyll content. With NaHS treatment, 3.24-fold and 2.28-fold increases were achieved in the maize leaves under –Fe and +Fe conditions compared with their control plants, respectively (Fig. 2B). Moreover, Fig. 2C showed that biomass under –Fe+NaHS was 2.12-fold higher than that of –Fe alone. Meanwhile, a 137% increase was also found under +Fe+NaHS than that of +Fe alone. In addition, it was found that H_2_S mediated the increase in chlorophyll content in a dose-dependent manner. For example, the chlorophyll content in +Fe plants treated with various concentrations of NaHS (10–1000 μM) were significantly higher than that of control plants, in which the optimal concentration of NaHS was 100 μM (Fig. 3).

### H_2_S-mediated chlorophyll increase is dependent on iron concentration

**Fig. 4.**
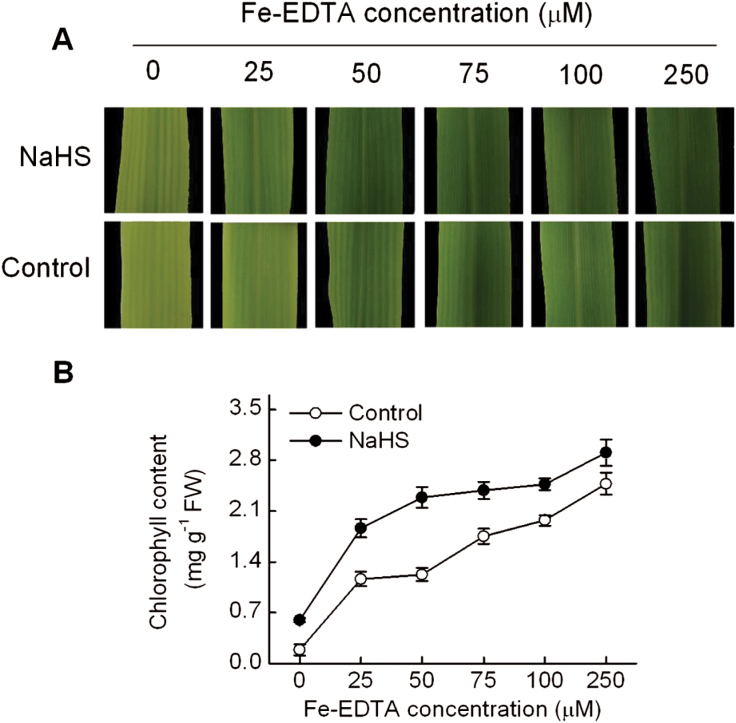
NaHS enhances the content of chlorophyll in maize seedlings grown under different Fe(III)-EDTA supplies. Maize seedlings were pre-treated with 100 µM NaHS for 8 d and then grown hydroponically in a nutrient solution with 0, 25, 50, 75, 100, or 250 µM Fe(III)-EDTA for 12 d.(A) Representative photographs of the fourth leaves of plants grown at different Fe(III)-EDTA concentrations. (B) Chlorophyll content in the fourth leaves. Data are presented as means ± SE. A colour version of this figure is available at *JXB* online.

To assess the relationship between H_2_S-mediated greening and iron availability, chlorophyll content of maize plants grown at different Fe(III)-EDTA concentrations (0–250 μM) plus 100 μM NaHS was analysed. Figure 4A shows representative leaves of plants grown in the above conditions. H_2_S prevented iron deficiency-induced chlorosis even in plants growing in the absence of iron by increasing the chlorophyll content three-fold compared with that of the control plants (Fig. 4B). Chlorophyll content in untreated maize leaves markedly increased with increasing concentrations of iron in the nutrient solution. Thus, between 0 and 250 μM Fe-EDTA, chlorophyll content increased from 0.18 to 2.48mg g^−1^ fresh weight. In H_2_S-treated plants, chlorophyll content increased from 0.59 to 2.90mg g^−1^ fresh weight for the same range of iron concentrations (Fig. 4B). In fact, the chlorophyll content of 100 μM Fe-EDTA-treated plants without H_2_S treatment (control plants) was the same level as that of plants grown in 25 μM Fe-EDTA treated with H_2_S. Plants grown under severe iron deficiency showed slow growth and usually fail to complete their vegetative cycle, however, H_2_S-treated plants exhibited normal development under –Fe conditions (Supplementary Fig. S1, available at *JXB* online).

### H_2_S treatment increases iron accumulation in maize plants

To evaluate whether H_2_S promotes an increase in iron accumulation inside the plant, total iron content in maize plants was estimated by ICP-MS. The iron concentration in the H_2_S-treated plants was significantly higher than that of the controls (the plants without H_2_S treatment) except for stems grown under 50 μM Fe-EDTA (Table 1). For example, more than a two-fold increase in H_2_S-treated roots of maize plants grown under 50 μM Fe-EDTA conditions was found. Indeed, iron concentration in H_2_S-treated leaves increased by 21% compared with the controls. Overall, H_2_S-treated leaves and roots have higher iron content, suggesting that H_2_S functions mainly through improving iron uptake and availability inside the leaf.

**Table 1.** Iron content in leaves, stems, and roots of maize plants Maize seedlings were pre-treated with 100 µM NaHS for 8 d and then grown in a nutrient solution containing 1 µM Fe(III)-EDTA or 50 µM Fe(III)-EDTA for 12 d.

	–Fe	–Fe+NaHS	+Fe	+Fe+NaHS
	Fe content (mg g^−1^ DW)			
Leaves	0.137±0.016 a	0.166±0.006 b	0.180±0.001 c	0.218±0.002 d
Stems	0.174±0.002 a	0.209±0.013 b	0.222±0.012 b	0.215±0.010 b
Roots	0.424±0.020 a	0.481±0.005 b	1.115±0.046 c	2.270±0.038 d

Data are presented as means ± SE.

Different letters indicate significant differences at *P*<0.05 from different treatments.

DW, dry weight; –Fe, 1 μM Fe; –Fe+NaHS, seedlings were pre-treated with 100 µM NaHS and then treated with 1 µM Fe; +Fe, 50 µM Fe; +Fe+NaHS, seedlings were pre-treated with 100 µM NaHS and then treated with 50 µM Fe.

### H_2_S regulates the expression of Fe homeostasis-related genes to iron acquisition in maize plants

**Fig. 5.**
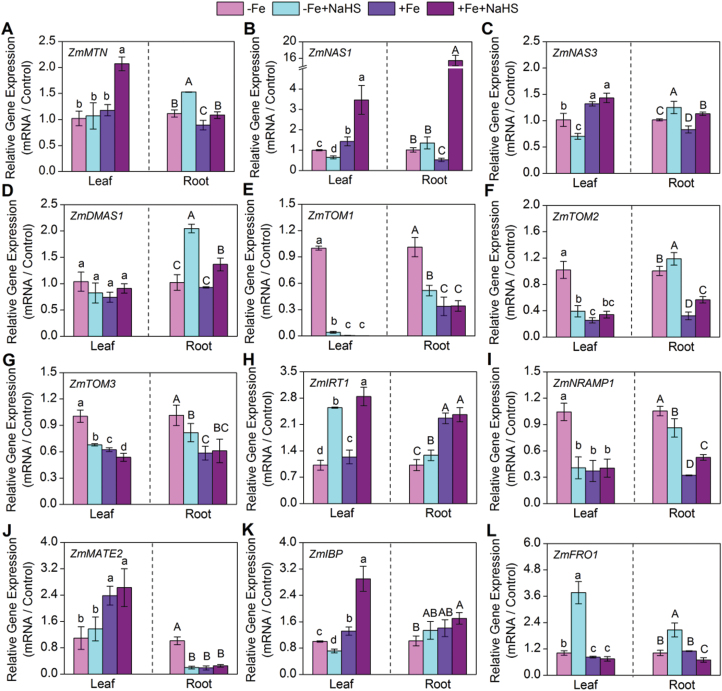
Gene expression of *ZmMTN* (A), *ZmNAS1* (B), *ZmNAS3* (C), *ZmDMAS1* (D), *ZmTOM1* (E), *ZmTOM2* (F), *ZmTOM3* (G), *ZmIRT1* (H), *ZmNRAMP1* (I), *ZmMATE2* (J), *ZmIBP* (K), and *ZmFRO1* (L) of maize leaves (left column) and roots (right column) with different treatments. Maize seedlings were pre-treated with 100 µM NaHS for 8 d and then grown in a nutrient solution containing 1 µM Fe(III)-EDTA or 50 µM Fe(III)-EDTA for 12 d. The relative mRNA level of each gene was normalized to the mRNA of *Zmactin2*. Data are presented as means ± SE of three replicates. Columns labelled with different letters indicate significant differences at *P*<0.05. –Fe, 1 µM Fe; –Fe+NaHS, seedlings were pre-treated with 100 µM NaHS and then treated with 1 µM Fe; +Fe, 50 µM Fe; +Fe+NaHS, seedlings were pre-treated with 100 µM NaHS and then treated with 50 µM Fe. A colour version of this figure is available at *JXB* online.

To investigate the physiological role of H_2_S on iron bioavailability, the expression of Fe homeostasis-related genes—those that encode enzymes involved in the MAs biosynthesis pathway, including the methionine cycle, as well as those for transcription factors and transporters involved in Fe homeostasis—was measured. Firstly, the gene expression of *ZmMTN* was markedly elevated in roots under H_2_S-treated seedlings (Fig. 5A). In addition, the expression levels of those genes involved in MAs biosynthesis including *ZmNAS1*, *ZmNAS3*, and *ZmDMAS1* were significantly induced by H_2_S under –Fe and +Fe conditions in roots of maize seedlings, but this function of H_2_S was not marked in leaves (Fig. 5B–D). Besides, transporter genes with similarities to *ZmTOM1* and *ZmTOM3* were induced in roots and leaves under the –Fe condition, but this induced expression was decreased due to the application of NaHS (Fig. 5E, G). In contrast, the expression of *ZmTOM2* was obviously higher in H_2_S-treated seedling roots than that of the controls (Fig. 5F). Moreover, the gene expression of *ZmIRT1* in leaves was up-regulated profoundly under H_2_S treatment, but 50 μM Fe-EDTA itself rather than NaHS treatment promoted the gene expression of *ZmIRT1* in roots (Fig. 5H). The expression levels of *ZmNRAMP1* and *ZmMATE2* in roots were induced by –Fe, but H_2_S could significantly inhibit this increase under the –Fe condition (Fig. 5I, J). Here, the gene expression of *ZmIBP* in +Fe+NaHS-treated maize leaves was increased 2.2-fold compared with the controls (+Fe), but the expression of this gene did not change significantly after NaHS treatment in roots (Fig. 5K). Finally, 3.7-fold and 2.0-fold increases of the gene expression of *FRO1* were found in H_2_S-treated leaves and roots of maize grown in –Fe solution compared with their controls, respectively. However, there was no obvious change between +Fe and +Fe+NaHS-treated maize leaves (Fig. 5L).

### H_2_S regulates the transcript abundance of *ZmYS1* and PSs secretion and content in maize seedling roots

**Fig. 6.**
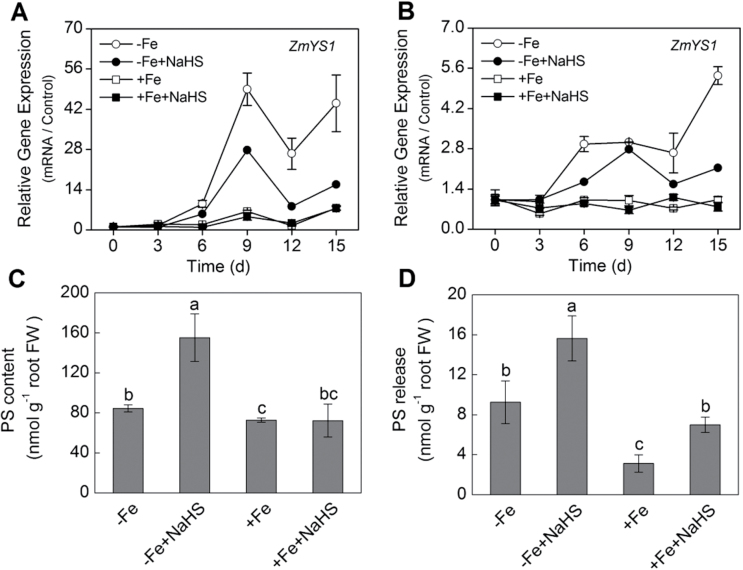
Time-dependent gene expression of *ZmYS1* in maize leaves (A) and roots (B). PSs accumulation in roots (C) and PSs release (D) of maize seedlings. Maize seedlings were pre-treated with 100 µM NaHS for 8 d and then grown in a nutrient solution containing 1 µM Fe(III)-EDTA or 50 µM Fe(III)-EDTA for 15 d. The relative mRNA level of each gene was normalized to the mRNA of *Zmactin2*. Data (A, B) are presented as means ± SE of three replicates. Data (C, D) are presented as means ± SE of four replicates. Columns labelled with different letters indicate significant differences at *P*<0.05. –Fe, 1 µM Fe; –Fe+NaHS, seedlings were pre-treated with 100 µM NaHS and then treated with 1 µM Fe; +Fe, 50 µM Fe; +Fe+NaHS, seedlings were pre-treated with 100 µM NaHS and then treated with 50 µM Fe.

To improve understanding of how H_2_S influences the response to Fe deficiency, the expression of *ZmYS1* in maize seedlings was analysed. The expression of *ZmYS1* in leaves and roots significantly increased with length of Fe-deficient treatment (Fig. 6A, B). The transcript abundance of *ZmYS1* in leaves after 9 d of Fe-deficient treatment increased 50-fold (Fig. 6A). Similarly, this gene expression in roots also increased by six-fold at 15 d after Fe-deficient treatment (Fig. 6B). However, *ZmYS1* transcript abundance in leaves and roots of maize seedlings treated with NaHS were significantly lower than those without NaHS treatment at each time point. In contrast to –Fe treatment, there was no significant difference between +Fe and +Fe+NaHS treatment in both leaves and roots of maize seedlings (Fig. 6A, B).

It is well known that secretion of PSs has been described as a physiological response to Fe deficiency in strategy II plants. After NaHS treatment, both the PSs accumulation in root tips and the rate of PSs exudation were evaluated. As shown in Fig. 6C, PSs accumulation in NaHS-treated roots was higher by up to two-fold compared with those without NaHS-treated roots under –Fe conditions, whereas no significant change was found between +Fe and +Fe+NaHS-treated roots. Similar to PSs accumulation, PSs release was also significantly induced by –Fe and NaHS (Fig. 6D). For instance, around a 1.7-fold increase of PSs release was observed in NaHS-treated maize roots under –Fe conditions. The same positive effect of NaHS on PSs release was confirmed in +Fe-treated maize roots.

### Effect of H_2_S on chloroplast ultrastucture in iron-deficient maize plants

**Fig. 7.**
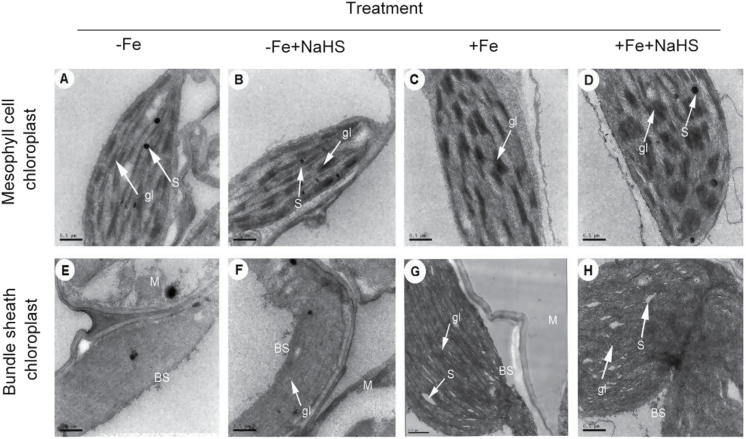
TEM analysis of the effect of NaHS on mesophyll cell (A–D) and bundle sheath (E–H) chloroplast ultrastructure of maize leaves.(A, E), –Fe; (B, F), –Fe+NaHS; (C, G), +Fe; (D, H), +Fe+NaHS; gl, grana lamella; S, starch; BS, bundle sheath cell; M, mesophyll cell. Bar = 0.5 µm.

Electron micrographs of mesophyll cells from iron-deficient maize plants revealed plastids with few photosynthetic lamellae and with some rudimentary grana, displaying typical features of thylakoid disorganization induced by iron deprivation (Fig. 7A). In contrast, when iron-deficient plants were treated with H_2_S, mesophyll chloroplasts appeared completely developed and the numbers of normal grana stacking and grana lamellae were significantly increased (Fig. 7B, D). For instance, the number of grana stacking in H_2_S-treated seedlings increased by 27% and 47% compared with that in the controls under –Fe or +Fe conditions by quantitative analysis, respectively (Supplementary Table S3, available at *JXB* online). Moreover, there was a two-fold increase in the number of grana lamellae both under –Fe+H_2_S and +Fe+H_2_S conditions compared with their controls (Supplementary Table S3, available at *JXB* online). In bundle sheath chloroplasts of iron-deficient plants, electron micrographs also revealed important differences between H_2_S-treated and control plants. Control plants (1 μM Fe-EDTA) had chloroplasts with no detectable starch granules and grana stacking, whereas chloroplast of H_2_S-treated plants showed slight grana stacking (Fig. 7F). However, under 50 μM Fe-EDTA treatment, pronounced starch granules and grana stacking was observed in chloroplasts. Furthermore, when treated with H_2_S, the number of starch granules and grana stacking increased slightly (Fig. 7G, H).

### Effect of H_2_S on endogenous H_2_S, GSH, and NPTs content in iron-deficient maize plants

A high accumulation of endogenous H_2_S in maize seedling leaves and roots caused by exogenously applied NaHS was observed under –Fe or +Fe conditions (Table 2). Meanwhile, to study further whether exogenously applied NaHS has an effect on thiol redox modification of proteins involved in iron homeostasis, GSH and NPTs content was measured. As shown in Table 2, NaHS treatment resulted in different degrees of increases in GSH and NPTs content in roots and leaves under –Fe or +Fe conditions.

**Table 2.** Effects of H_2_S donor NaHS and iron on endogenous H_2_S, NPTs, and GSH content in leaves and roots of maize seedlings Maize seedlings were pre-treated with 100 µM NaHS for 8 d and then grown in a nutrient solution containing 1 µM Fe(III)-EDTA or 50 µM Fe(III)-EDTA for 12 d.

Treatment	Endogenous H_2_S content (μmol g^−1^ FW)		NPTs (μmol g^−1^ FW)		GSH (nmol g^−1^ FW)	
	Leaf	Root	Leaf	Root	Leaf	Root
−Fe	0.022±0.005 a	0.032±0.007 A	1.58±0.13 a	8.23±0.32 A	162.8±29.6 a	407.7±1.95 A
−Fe+NaHS	0.069±0.006 b	0.064±0.005 B	1.92 0.27 a	10.66±0.69 B	372.1±27.5 b	534.3±48.4 B
+Fe	0.025±0.004 a	0.023±0.001 A	2.75±0.31 b	8.35±0.61 A	378.3±7.71 b	476.2±34.4 B
+Fe+NaHS	0.108±0.006 c	0.100±0.015 C	4.12±0.27 c	11.30±1.53 B	619.2±97.6 c	510.3±42.4 B

Data are presented as means ± SE.

Different letters indicate significant differences at *P*<0.05 from different treatments.

–Fe, 1 μM Fe; –Fe+NaHS, seedlings were pre-treated with 100 µM NaHS and then treated with 1 µM Fe; +Fe, 50 µM Fe; +Fe+NaHS, seedlings were pre-treated with 100 µM NaHS and then treated with 50 µM Fe.

FW, fresh weight.

### H_2_S regulates the expression of sulphur metabolism-related genes in iron-deficient maize plants

**Fig. 8.**
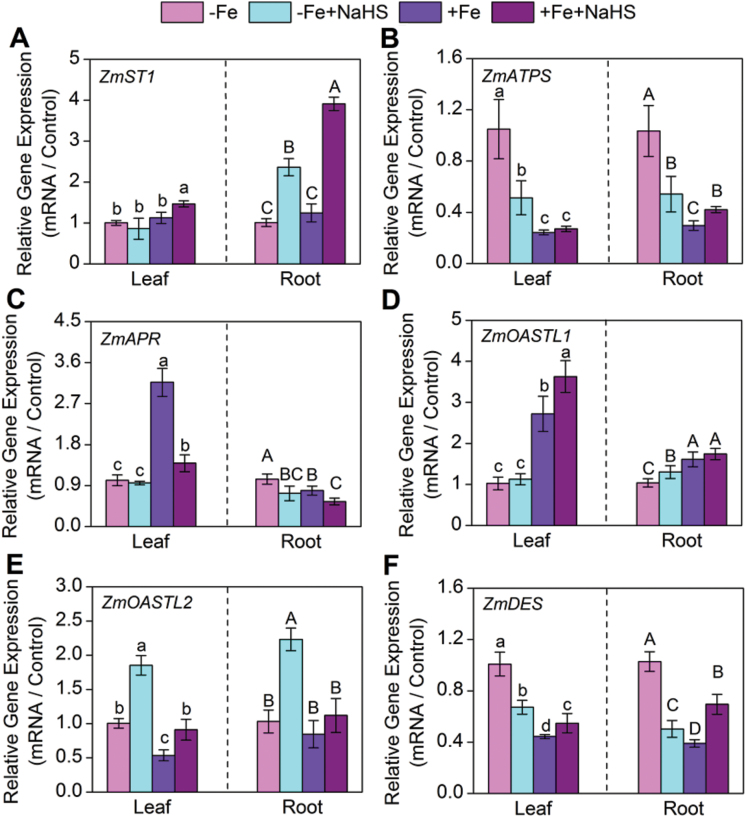
Gene expression of *ZmST1* (A), *ZmATPS* (B), *ZmAPR* (C), *ZmOASTL1* (D), *ZmOASTL2* (E), and *ZmDES* (F) of maize leaves (left column) and roots (right column) with different treatments. Maize seedlings were pre-treated with 100 µM NaHS for 8 d and then grown in a nutrient solution containing 1 µM Fe(III)-EDTA or 50 µM Fe(III)-EDTA for 12 d. The relative mRNA level of each gene was normalized to the mRNA of *Zmactin2*. Data are presented as means ± SE of three replicates. Columns labelled with different letters indicate significant differences at *P*<0.05. –Fe, 1 µM Fe; –Fe+NaHS, seedlings were pre-treated with 100 µM NaHS and then treated with 1 µM Fe; +Fe, 50 µM Fe; +Fe+NaHS, seedlings were pre-treated with 100 µM NaHS and then treated with 50 µM Fe. A colour version of this figure is available at *JXB* online.

Under NaHS treatment, the amount of *ZmST1* transcript encoding a high-affinity sulphate transporter in maize seedling roots increased by 134% and 215% under –Fe and +Fe conditions, respectively (Fig. 8A). However, in leaves, there was no obvious change in the amount of *ZmST1*. In contrast, exogenously applied NaHS could significantly down-regulate the amount of *ZmATPS* transcript under iron-deficient maize seedling leaves and roots (Fig. 8B). Similarly, the amount of *ZmAPR* transcript in iron-deficient maize roots also was decreased (Fig. 8C). Interestingly, the amount of *ZmOASTL1* and *ZmOASTL2* transcripts encoding *O*-acetyl-L-serine(thiol)lyase was significantly increased under the –Fe+NaHS condition compared with that of the –Fe condition in maize seedling roots (Fig. 8D, E). Moreover, the expression of *ZmOASTL2* gene in –Fe or +Fe maize seedling leaves was up-regulated by exogenously applied NaHS (Fig. 8E). On the contrary, under the –Fe condition NaHS also decreased significantly the amounts of *ZmDES* transcript encoding cysteine desulfhydrase in leaves and roots, but under the +Fe condition, its expression was up-regulated by NaHS in leaves and roots (Fig. 8F).

### Effect of H_2_S on photosynthesis in iron-deficient maize plants

**Fig. 9.**
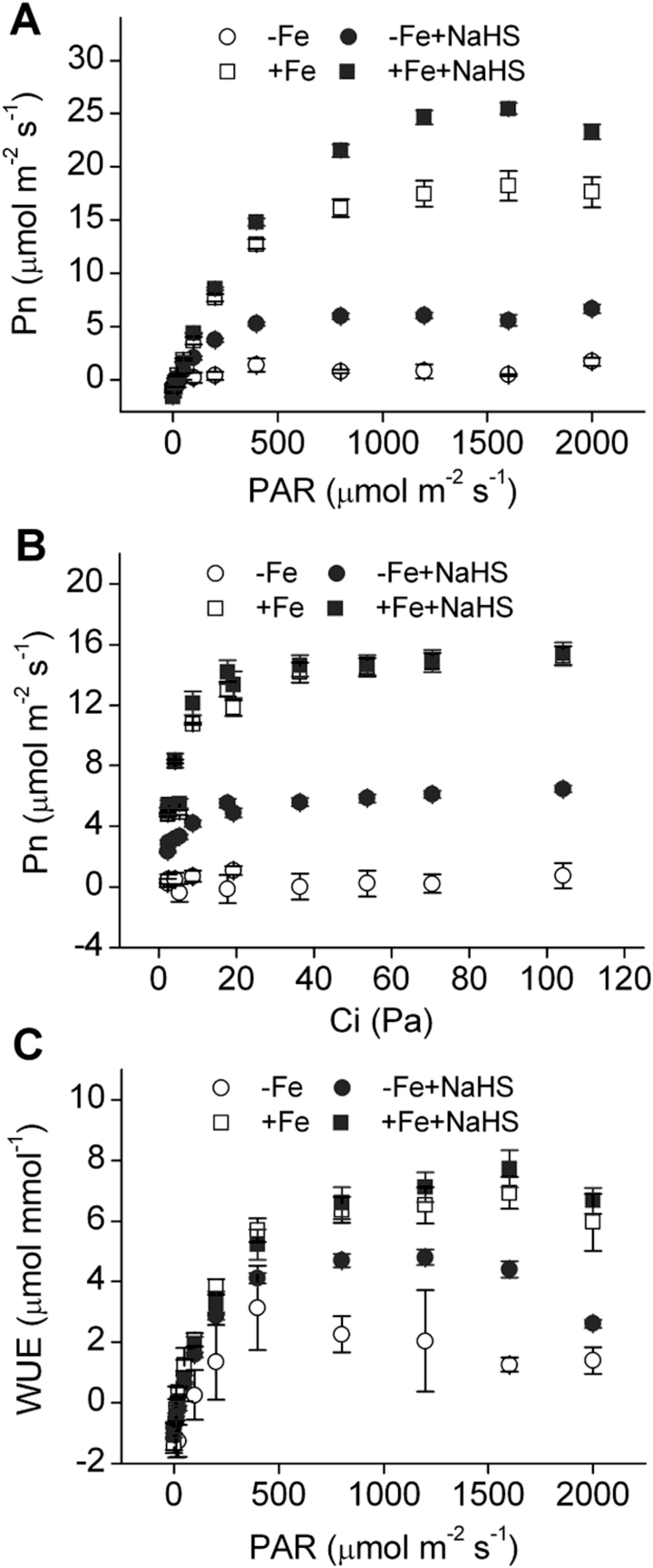
Light-response curve (A), Intercellular CO_2_-response curve (B), and WUE-response curve (C) of maize plants grown under iron-deficient conditions. Maize seedlings were pre-treated with 100 µM NaHS for 8 d and then grown in a nutrient solution containing 1 µM Fe(III)-EDTA or 50 µM Fe(III)-EDTA for 12 d. Data are presented as means ± SE. –Fe, 1 µM Fe; –Fe+NaHS, seedlings were pre-treated with 100 µM NaHS and then treated with 1 µM Fe; +Fe, 50 µM Fe; +Fe+NaHS, seedlings were pre-treated with 100 µM NaHS and then treated with 50 µM Fe.

Iron deficiency causes a marked reduction in photosynthesis. Therefore, photosynthesis was further measured in iron-deficient maize plants. Light-response curves and CO_2_-response curves indicated that Pn was obviously higher in NaHS-treated maize plants than in the controls under –Fe or +Fe conditions (Fig. 9A, B). Similarly, water use efficiency (WUE) was also increased in NaHS-treated maize plants, particularly under the –Fe condition (Fig. 9C). In addition, the *R*d, *L*cp, *L*sp, *AQE*, and *P*max of maize plants were calculated by modelling the response of leaf Pn to PAR by a non-rectangular hyperbola and the *CE* was acquired by a rectangular hyperbola (Table 3). *R*d, *L*sp, *AQE*, *P*max, and *CE* were notably higher under NaHS treatment than their controls. Meanwhile, these parameters were obviously higher under +Fe treatment compared with those under –Fe treatment, except for *L*cp. Collectively, these results suggested that maize photosynthetic characteristics were significantly affected by NaHS treatment.

**Table 3.** *Effects of H*_*2*_ *S donor NaHS and iron on* AQE, R*d,* L*cp,* L*sp,* P*max, and* CE *of maize seedlings*

Variables	–Fe	–Fe+NaHS	+Fe	+Fe+NaHS
*AQE*	0.014±0.002a	0.038±0.004 b	0.051±0.003 c	0.050±0.001 c
*R*d (μmol CO_2_ m^−2^ s^−1^)	0.440±0.255 a	0.989±0.052 b	0.887±0.159 b	1.040±0.050 b
*L*cp (μmol m^−2^ s^−1^)	34.80±9.14 a	26.43±1.87 a	17.16±2.17 b	20.73±0.84 c
*L*sp (μmol m^−2^ s^−1^)	214.5±80.4 a	226.7±16.2 a	416.7±46.7 b	562.3±7.86 c
*P*max (μmol CO_2_ m^−2^ s^−1^)	2.74±0.71 a	7.48±0.51 b	20.20±1.78 c	27.17±0.66 d
*CE* (mol m^−2^ s^−1^)	0.036±0.024 a	0.730±0.184 b	2.819±0.055 c	5.686±1.082 d

Data are presented as means ±SE.

Different letters indicate significant differences at *P*<0.05 from different treatments.

–Fe, 1 μM Fe; –Fe+NaHS, seedlings were pre-treated with 100 µM NaHS and then treated with 1 µM Fe; +Fe, 50 µM Fe; +Fe+NaHS, seedlings were pre-treated with 100 µM NaHS and then treated with 50 µM Fe.

Effect of H_2_S on the protein expression of RuBISCO LSU and PEPC in iron-deficient maize plants

**Fig. 10.**
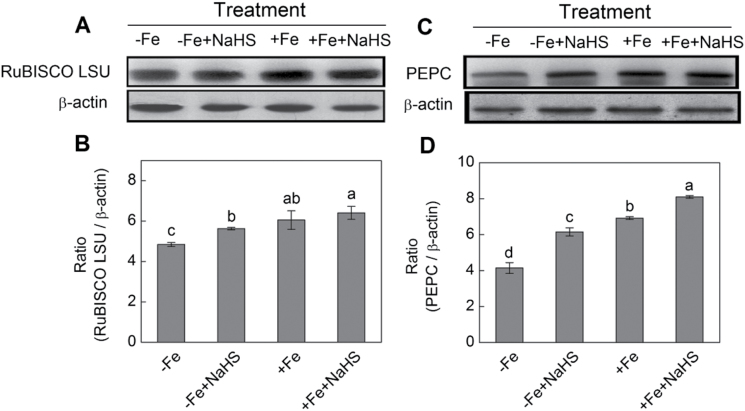
Western blot analysis of RuBISCO LSU (A) and PEPC (C) of maize plants. Maize seedlings were pre-treated with 100 µM NaHS for 8 d and then grown in a nutrient solution containing 1 µM Fe(III)-EDTA or 50 µM Fe(III)-EDTA for 12 d. Relative expression level is shown as the ratio of RuBISCO LSU:β-actin (B) and PEPC:β-actin (D) using Quantity One software. Data are presented as means ± SE. Columns labelled with different letters indicate significant differences at *P*<0.05. –Fe, 1 µM Fe; –Fe+NaHS, seedlings were pre-treated with 100 µM NaHS and then treated with 1 µM Fe; +Fe, 50 µM Fe; +Fe+NaHS, seedlings were pre-treated with 100 µM NaHS and then treated with 50 µM Fe.

As shown in Fig. 10A, B, after quantification and normalization to β-actin, the protein expression of RuBISCO LSU showed a slight increase in the leaves of maize grown in the –Fe condition after NaHS treatment. However, there was no significant effect of NaHS on RuBISCO LSU protein expression under high iron supply conditions. Meanwhile, it was also found that the protein expression of PEPC was significantly enhanced by H_2_S compared with that of their controls (Fig. 10C, D).

### Effect of H_2_S on *ZmRBCL*, *ZmRBCS*, *ZmpsbA*, and *ZmPEPC* gene expression in iron-deficient maize plants

**Fig. 11.**
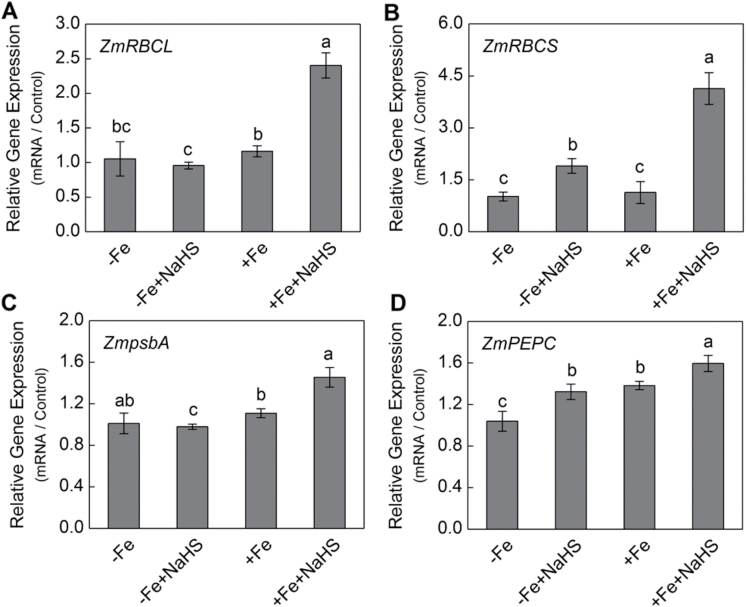
Gene expression of *ZmRBCL* (A), *ZmRBCS* (B), *ZmpsbA* (C), and *ZmPEPC* (D) of maize leaves. Maize seedlings were pre-treated with 100 µM NaHS for 8 d and then grown in a nutrient solution containing 1 µM Fe(III)-EDTA or 50 µM Fe(III)-EDTA for 12 d. The relative mRNA level of each gene was normalized to the mRNA of *Zmactin2*. Data are presented as means ± SE of three replicates. Columns labelled with different letters indicate significant differences at *P*<0.05. –Fe, 1 µM Fe; –Fe+NaHS, seedlings were pre-treated with 100 µM NaHS and then treated with 1 µM Fe; +Fe, 50 µM Fe; +Fe+NaHS, seedlings were pre-treated with 100 µM NaHS and then treated with 50 µM Fe.

Iron deficiency causes a marked reduction in the accumulation of chloroplastic proteins and mRNAs. Two-fold higher gene expression of *ZmRBCL* was detected in NaHS-treated maize than that of the control under +Fe conditions, although there was no obvious change between NaHS-treated maize and control plants under –Fe conditions (Fig. 11A). Similarly, under NaHS-treatment conditions, the relative gene expression of *ZmRBCS* in leaves of maize under –Fe and +Fe conditions was two-fold and four-fold higher compared with their controls, respectively (Fig. 11B). In addition, under +Fe conditions, *ZmpsbA* expression increased by 31% in H_2_S-treated plants compared with the control (Fig. 11C). Moreover, *ZmPEPC* expression increased by 28% and 16% in H_2_S-treated plants both under –Fe and +Fe conditions compared with their controls, respectively (Fig. 11D).

## Discussion

It is well known that a high concentration of iron is required to maintain the structural and functional integrity of thylakoid membranes in chloroplasts, because more than 80% of leaf iron is located in the chloroplast (Terry and Abadia, 1986; Kim and Guerinot, 2007). Chlorophyll constitutes the major component of chloroplasts and is positively correlated with photosynthesis and leaf iron concentration. In the present study, the effects of H_2_S on the expression of genes and proteins related to photosynthesis, sulphur metabolism, and iron acquisition were investigated in maize plants. The results showed that exogenous H_2_S is closely related to iron uptake, transport, and accumulation, and consequently promotes chlorophyll biosynthesis, chloroplast development, and photosynthesis in plants.

Under iron-deficient conditions, H_2_S increased the chlorophyll content in maize leaves by three-fold over control plants, achieving a similar chlorophyll level to that found in maize plants grown under iron-sufficient conditions. H_2_S-mediated chlorophyll increase was accompanied by the accumulation of transcripts of genes including *ZmpsbA* of PSII, *ZmPEPC*, *ZmRBCL*, and *ZmRBCS*. Previous reports showed that levels of these gene transcripts were reduced under iron deficiency and would be recovered after iron supply (Spiller *et al.*, 1987). The results presented here are consistent with observations by other researchers showing that NO and CO improve iron availability in plants (Graziano *et al.*, 2002; Kong *et al.*, 2010).

### H_2_S regulates chlorophyll biosynthesis and chloroplast development in iron-deficient plants

Previous studies have indicated that iron deficiency could cause an interveinal yellowing of the leaves, the disruption of chloroplast ultrastructure, and degradation of chloroplast protein components (Terry, 1980; Winder and Nishio, 1995; Thoiron *et al.*, 1997). Indeed, several steps involved in photosynthetic pigment synthesis and chloroplast ultrastructure establishment are dependent on iron availability (Briat *et al.*, 2007). In the present study, maize seedlings under iron-deficient conditions displayed severe chlorotic characteristics with an interveinal yellowing and a low concentration of chlorophyll (Figs 1–4). Similarly, a typical phenotype of chlorosis in *Arabidopsis* and *Chlamydomonas reinhardtii* grown in an iron-deficient solution has been reported, which resulted in the reduction of photosynthetic rate (Briat *et al.*, 2007; Kong *et al.*, 2010). However, exogenously applied H_2_S donor NaHS rather than other sulphur- or sodium-containing compounds increased the chlorophyll content in iron-deficient maize seedlings (Fig. 1), which is consistent with the observations by other researchers that H_2_S affects chlorophyll content (Zhang *et al.*, 2009; Chen *et al.*, 2011). Moreover, the ultrastructure of chloroplasts from mesophyll cells was severely altered under iron deficiency conditions. As reported previously, chloroplasts exhibited few photosynthetic lamellae and granain iron-deficient mesophyll cells (Fig. 7A, C) (Stocking, 1975; Thoiron *et al.*, 1997; Muneer *et al.*, 2014). Interestingly, chloroplasts from mesophyll cells of H_2_S-treated plants developed extensive grana and increased the number of grana lamellae as well (Fig. 7B, D). Similarly, Muneer *et al.* (2014) reported that Fe-deficient results in a reduction of chloroplast number, and the disintegration of the lamellar network inside the chloroplast, most severely in the absence of sulphur. However, the context of their experiments was slightly different; for example, here, changes in the number of chloroplasts under –Fe and +Fe conditions were not assessed. Moreover, this study focused on the signalling function of exogenously applied NaHS in terms of Fe-deficiency coping strategies. These results showed that H_2_S, as a signalling molecule, could promote the biogenesis of chloroplasts by increasing the number of grana lamellae and the biosynthesis of chlorophyll, which will lead to an enhancement of photosynthesis in iron-deficient maize plants.

### H_2_S increases iron accumulation by changing of iron homeostasis-related genes and *ZmYS1* expression, PSs secretion, and PSs content in iron-deficient plants

Some iron homeostasis-regulated genes play a crucial role in iron uptake and translocation in plants (Hell and Stephan, 2003). Results presented here showed that NaHS improved significantly iron accumulation in plant tissues under iron-deficient conditions (Table 1). These results are in agreement with previous studies that revealed CO could increase iron accumulation in both *Arabidopsis* root and leaf tissues and *Chlamydomonas reinhardtii* cells (Kong *et al.*, 2010). Therefore, it was concluded that H_2_S can confer iron homeostasis through either intracellular iron mobility or accumulation in plants.

The analysis of iron homeostasis-related genes expression would allow a better understanding of iron deficiency-induced chlorosis and the role of H_2_S in iron acquisition and chloroplast development. In this respect, results showed that NaHS could regulate the expression of *ZmMTN*, *ZmNAS1*, *ZmNAS3*, and *ZmDMAS1* in roots of iron-deficient maize seedlings and then increase the methionine and nicotianamine (NA) contents for coping with iron deficiency (Fig. 5A–D). Similarly, the expression of *ZmTOM1*, *ZmTOM2*, and *ZmTOM3* also were induced by iron deficiency, but exogenously applied NaHS could attenuate the increase of iron deficiency-induced expression (Fig. 5E–G). Besides, previous studies showed that once Fe(III) is reduced to Fe(II) and transported into plants, iron-regulated transporter (*IRT*) is responsible for iron uptake in roots (Vert *et al.*, 2001; Vert *et al.*, 2002). Research presented here indicated that the abundance of *ZmIRT1* was up-regulated by H_2_S in leaves or roots of maize plants grown under –Fe and +Fe conditions, suggesting that H_2_S may mediate iron acquisition through the activation of *ZmIRT1* gene expression in iron-stressed plants (Fig. 5H). Similarly, some studies have demonstrated that NO and CO can significantly enhance the gene expression of *IRT1* in iron-deficient plants (Graziano and Lamattina, 2007; Kong *et al.*, 2010). In plants, IBP (ferritin) is a large polymeric protein that can store up to 4000 atoms of iron within its protein shell and be capable of releasing iron when required (Briat *et al.*, 1999). Ferritins are mainly located in chloroplast and accumulate when iron is sufficiently available (Briat *et al.*, 1999). Here, the expression abundance of *ZmIBP* (IBP) was also found to be up-regulated by H_2_S in leaves or roots of maize plants grown under –Fe and +Fe conditions (Fig. 5K). A previous study also indicated that NO promoted accumulation of both ferritin mRNA and protein in *Arabidopsis* (Murgia *et al.*, 2002). Indeed, results presented here showed that *ZmFRO1* gene expression in leaf and root of maize plants grown under the –Fe condition was up-regulated by H_2_S treatment. However, under the +Fe condition, no obvious regulation effect of H_2_S was found (Fig. 5L). Graziano and Lamattina (2007) also found that NO could up-regulate the mRNA expression of *LeFRO1* in tomato grown under –Fe conditions, but had almost no effect on this gene expression in tomato grown under high iron levels. Likewise, Chen W. *et al.* (2010) have also reported that both auxin (NAA) and NO could enhance the expression of *AtFRO2* in *Arabidopsis* grown under iron-deficient conditions, but almost no effect under high levels of iron. Moreover, a previous study has reported that there is a positive correlation between *FRO1* gene expression and ferric-chelate reductase activity in iron-deficient roots (Connolly *et al.*, 2003). Interestingly, under –Fe conditions, the expression of *ZmFRO1* and *ZmIRT1* in maize leaves and roots are increased by addition of NaHS. These results are consistent with Zuchi *et al.* (2009), who reported that Fe deficiency and S-sufficiency caused increases in the expression levels of *LeIRT1* and *LeFRO1* in tomato seedlings. However, it was noteworthy that the expression of *ZmFRO1*, *ZmIBP*, and *ZmIRT1* in –Fe plants is very similar to that of +Fe plants, especially in leaves, although these genes are generally induced by Fe deficiency. Moreover, Paolacci *et al.* (2014) reported that the expression of *SlIRT1* and *SlFRO1* was strongly up-regulated by iron deprivation in roots of tomato seedlings. However, in the present study, the expression of *ZmIRT1* was down-regulated by iron deprivation in leaves and roots of maize seedlings.These results are contradictory with previous studies. The following explanations are presented: Firstly, the expression of *FRO1*, *IBP*, and *IRT1* mainly functioned in dicotyledons and their expression levels were up-regulated under –Fe conditions (Meiser *et al.*, 2011; Nishida *et al.*, 2011). However, there was little focus on these gene expressions in gramineous plants, such as maize. Therefore, it can be speculated that the response of *FRO1*, *IBP*, and *IRT1* gene expression is different between dicotyledons and gramineous plants. Secondly, previous studies showed that 50 μM Fe-EDTA represents iron-insufficient conditions for the growth of maize seedlings (Stocking, 1975; Graziano *et al.*, 2002). Thirdly, different plants respond differently to the effects of iron concentration, which has a close relationship with plant growth environment and the time of iron treatment. Fourthly, Gonzalo *et al.*, (2011) showed that the gene expression of *FRO2* and *IRT1* was up-regulated after 5 d of iron-shortage, but their expression level was down-regulated after 6 d of iron-shortage compared with iron-sufficient conditions in *Prunus* plants. These results concur with those presented here relating to gene expression of *ZmFRO1*, *ZmIBP*, and *ZmIRT1* in iron-deficient maize plants, which have a close relationship with the plant growth environment, time of iron treatment, and the concentration of iron treatment. Finally, importantly, Graziano and Lamattina (2007) reported that in leaves, *LeFRO1* expression does not depend on the iron status, there being no changes between iron-deficient and iron-sufficient conditions. This result confirmed that the regulation of *LeFRO1* in leaves is different from that in roots.

Iron acquisition in gramineous plants is characterized by secretion of chelating compounds such as PSs; PSs and Fe(III) then form a complex [Fe(III)-PS], which is subsequently transported by a specific Fe(III)-PS transporter in plant roots (Astolfi *et al.*, 2012). Results presented here showed that NaHS could enhance iron assimilation through increasing PSs content and PSs secretion in roots of maize under –Fe and +Fe conditions (Fig. 6C, D). Moreover, PSs biosynthesis requires methionine, therefore the effect of NaHS on Fe acquisition could be presumably due to an enhanced assimilation of sulphur and its subsequent incorporation into methionine in order to sustain an increased production of PSs and NA. The most well characterized Fe-PS transporter is the maize oligo peptide transporter (OPT) family membrane, *ZmYS1*. *ZmYS1* is expressed in roots in response to iron deficiency and re-supplying iron decreases the expression of *ZmYS1* (Morrissey and Guerinot, 2009). In the present study, although NaHS increased PSs content and PSs secretion in roots of maize plants under –Fe conditions (Fig. 6C, D), the expression level of *ZmYS1* was reduced by NaHS treatment in roots of maize plants (Fig. 6B). Besides, a baseline for *ZmYS1* gene expression in iron-deficient maize seedling roots after the NaHS treatment and before Fe treatment was established. Results showed that *ZmYS1* gene expression was significantly decreased after exogenously applied NaHS compared with the controls under iron-deficient conditions. Interestingly, when 1 μM Fe was added to the solution, *ZmYS1* gene expression also was decreased (Supplementary Fig. S2, available at *JXB* online). Similarly, Astolfi *et al.* (2010) reported that *HvYS1* transcript abundance was progressively decreased when the S- and Fe-deficient plants were supplied with both nutrients. One possible explanation for this disagreement is that the decreased expression level of *ZmYS1* prompted NaHS to alleviate the symptoms of iron deficiency and negating the need for an increase in the expression of this transporter. On the other hand, the enhancement of PSs secretion and PSs accumulation in root by NaHS treatment could increase the mobility of Fe(III) by the form of PS-Fe(III) in plant root and rhizosphere. Taken together, these results suggested that H_2_S is a necessary signal molecule for the expression of iron homeostasis-related genes and PSs secretion and accumulation in maize plants under –Fe and +Fe conditions.

### H_2_S could regulate sulphur-containing metabolites and sulphur metabolism-related genes expression to cope with iron deficiency in maize seedlings

Sulphate assimilation is an energy demanding process and supplying H_2_S would directly feed into cysteine and GSH synthesis. Previous studies have shown that H_2_S exposure generally results in an increased content of water-soluble NPT compounds including GSH and cysteine in shoot, and in some species, an increase in the sulphate content of the shoot has been observed (Schutz *et al.*, 1991; Durenkamp and De Kok, 2004; Riemenschneider *et al.*, 2005*b*; Durenkamp *et al.*, 2007). In this study, a high accumulation of endogenous H_2_S in maize seedling leaves and roots caused by exogenously applied NaHS was observed under –Fe or +Fe conditions (Table 2). Meanwhile, NaHS treatment resulted in different degrees of increases in GSH and NPTs contents in roots and leaves under –Fe or +Fe conditions. These results indicated that exogenously applied NaHS was not only directly fed into cysteine and GSH synthesis by regulating sulphur metabolism-related enzyme activities and gene expression, but also increased the content of endogenous H_2_S in plants. Interestingly, a previous paper also showed that externally applied NaHS led to increases in the internal concentration of H_2_S, the contents of GSH, NPTs and cysteine in *S. oleracea* leaves by affecting the enzyme activities of OAS-TL and l-cysteine desulphydrase (LCD) (Chen *et al.*, 2011). The pathway of sulphur assimilation in plants is divided into two reaction sequences: sulphate reduction and cysteine synthesis. Sulphate reduction is preceded by the uptake of sulphate from the soil into plant by specific sulphate transporters (Buchner *et al.*, 2004). Within the plant, sulphate is firstly activated by ATP sulphurylase (ATPS), and the resulting adenosine-5′-phosphosulphate (APS) is reduced in a two-step reaction via sulphite to sulphide by APS reductase (APR) and sulphide reductase (SiR). Sulphide is then incorporated into OAS by OAS-(thiol)lyase (OAS-TL) to form cysteine, which serves as a sulphur source for all organic molecules containing reduced sulphur, e.g. GSH, protein, cofactors, and vitamins (Takahashi *et al.*, 2011). Therefore, expression of the sulphur metabolism-related genes was analysed in iron-deficient maize seedling leaves and roots. Results showed that under –Fe or +Fe conditions, exogenously applied NaHS could affect the expression levels of sulphate transporter 1 (ST1) (*ZmST1*) gene and sulphate reduction-related genes including *ZmATPS* and *ZmAPR* in maize seedlings (Fig. 8A–C). Moreover, the amounts of the transcript of *ZmOASTL1* and *ZmOASTL2* encoding *O*-acetyl-L-serine(thiol)lyase were significantly increased under –Fe+NaHS conditions compared with –Fe conditions in maize seedling roots (Fig. 8D, E). The expression of *ZmOASTL2* gene in –Fe or +Fe maize seedling leaves was up-regulated by exogenously applied NaHS (Fig. 8E). These results were consistent with previous studies. For instance, Chen *et al.* (2011) reported that exogenously applied NaHS could up-regulate the expression of *OASTL* gene in *S. oleracea* seedling leaves. Birke *et al.* (2015) reported that H_2_S exposure down-regulated the expression levels of *ATPS1*, *APR2*, and *DES1*, but up-regulated the expression levels of *S*-sulphocysteine synthase (CS26) in tomato seedling leaves. Therefore, it was concluded that H_2_S as a signalling molecule could cope with iron deficiency through increasing sulphur-containing metabolites including GSH and NPTs and regulating the expression level of sulphur metabolism-related genes in maize seedlings. Interestingly, Astolfi *et al.* (2004, 2006*b*, 2010, 2012) reported that improved sulphur nutrition status enables plants to better cope with Fe deficiency. However, unlike previous studies, this study mainly focused on the signalling function of H_2_S in improving adaptation of maize seedlings to iron deficiency.

### H_2_S-improved photosynthesis (by increasing the protein and gene expression of photosynthetic enzyme) is dependent on iron availability

Iron deficiency can cause a decrease of chlorophyll (chlorosis) and then lead to the reduction of leaf photosynthetic rate (Larbi *et al.*, 2006). Moreover, other studies showed that the efficiencies of light absorption, PSII and RuBISCO carboxylation were significantly reduced under iron-deficient conditions (Stocking, 1975; Larbi *et al.*, 2006). Besides, chlorotic leaves caused by iron deficiency exhibited lower stomatal apertures and lower photosynthetic rates, finally resulting in the reduction of WUE (Larbi *et al.*, 2006). In the present study, light-response curves and CO_2_-response curves indicated that photosynthetic rate was significantly enhanced by H_2_S under –Fe and +Fe conditions, suggesting that H_2_S promotes photosynthesis through increasing iron acquisition in iron-stressed plants (Fig. 9A, B). Besides, WUE was also obviously enhanced by H_2_S (Fig. 9C). Meanwhile, further analysis of photosynthetic features revealed that *P*max was also significantly higher in NaHS-treated seedlings grown under –Fe and +Fe solution than that in their controls (Table 3). This result is important for clarifying the effect of H_2_S on photosynthesis by enhancing iron acquisition, because a higher *P*max allows seedlings to acquire a higher potential to assimilate carbon dioxide. *AQE* reflects the light use efficiency of plants at low light intensities. This data showed that *AQE* had an obvious increase in NaHS-treated maize plants grown in –Fe solution compared with the control. These results indicated that NaHS could enhance the light use efficiency of maize plants under –Fe conditions at a lower light intensity. *L*sp represents the capacity of plants to use the maximal light. The results showed that *L*sp was higher in NaHS-treated maize plants grown in +Fe solution than that in the control, but there was no obvious change in the –Fe solution, indicating that NaHS can increase the maximal light use efficiency of maize plants grown in +Fe solution under a high light intensity. Importantly, *CE* has been considered to play a major role in the improvement of photosynthesis (Pell *et al.*, 1992; Farage and Long, 1999). Data presented here showed that *CE* was significantly increased by H_2_S in maize plants grown in –Fe and +Fe solutions, suggesting that H_2_S could induce the enhancement in carboxylation process through increasing iron acquisition in iron-deficient plants (Table 3).

RuBISCO and PEPC are key enzymes controlling photosynthetic carbon fixation in plants, and the level of activated RuBISCO and PEPC are closely related to the *L*sp, *CE*, and the rate of photosynthetic carbon assimilation (Lin and Hsu, 2004). The present results showed that the protein expression of RuBISCO LSU and PEPC were significantly increased by H_2_S in maize plants grown under –Fe and +Fe conditions compared with their controls, suggesting that the improvement in photosynthesis with NaHS treatment might be a result of an increase in RuBISCO and PEPC levels (Fig. 10). Besides, results presented here showed that H_2_S-mediated chlorophyll increase and the enhancement of photosynthetic rate were accompanied by the accumulation of transcripts encoding the D1 protein of PSII (*ZmpsbA*), RuBISCO large subunit (*ZmRBCL*) and small subunit (*ZmRBCS*), and PEPC (Fig. 11). Previous studies showed that the levels of these transcripts were reduced during iron deficiency and recovered after iron supply (Spiller *et al.*, 1987). Similarly, Graziano *et al.* (2002) also reported that NO-mediated chlorophyll increase was accompanied by the accumulation of transcripts encoding both the D1 protein of PSII and the RuBISCO large subunit. Taken together, all the evidence presented here indicates that the positive effect of H_2_S on leaf photosynthesis and chlorophyll biosynthesis occurs mainly through stimulating the transcription of *ZmRBCL*, *ZmRBCS*, *ZmPEPC*, and *ZmpsbA* and enhancing the accumulation of RuBISCO and PEPC protein in maize grown under iron-deficient conditions.

### Pathway of H_2_S-improved adaptation of maize seedlings to iron deficiency

**Fig. 12.**
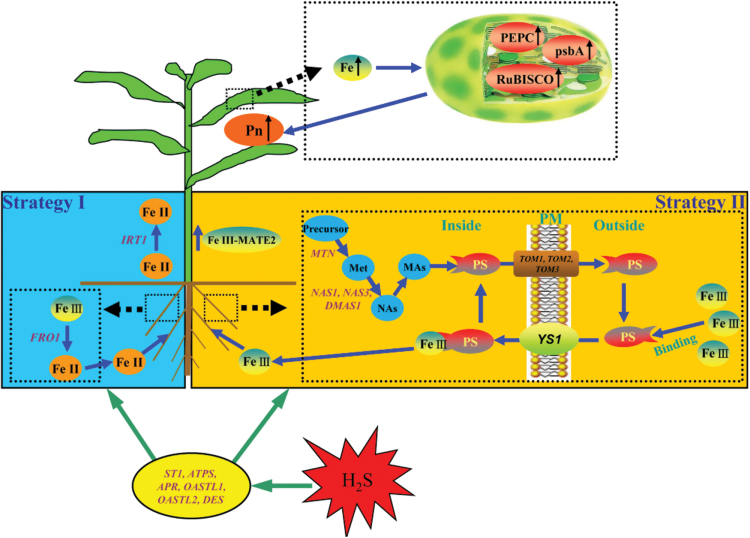
A schematic model for H_2_S-improved adaptation of maize seedlings to iron deficiency. ATPS, ATP sulphurylase; APR, APS reductase; DES, Cys desulfhydrase; DMAS1, deoxymugineic acid synthase 1; FRO1, ferric-chelate reductase 1; MATE2, multi-drug and toxin efflux 2; MTN, *S*-adenosyl homocysteine nucleosidase; NAS1/3, nicotianamine synthase 1/3; NAs, nicotianamine; OASTL1/2, *O*-acetyl-L-serine(thiol)lyase 1/2; psbA, D1 protein; ST1, sulphate transporter 1; TOM1/2/3, transporter of MAs 1/2/3; YS1, yellow stripe 1. A colour version of this figure is available at *JXB* online.

Based on results shown here and current knowledge about the mechanisms of plant response to iron deficiency, a signalling pathway by which H_2_S improves adaptation of maize seedlings to iron deficiency has been proposed. As shown in Fig. 12, H_2_S could regulate the expression of sulphur metabolism-related genes and further promote iron mobility and uptake through mainly the mechanism of strategy II—including increasing the secretion of PSs and PSs content in the roots of maize seedlings and changing the mRNA expression abundance of *ZmYS1* and iron homeostasis-related genes. Besides, it was hypothesized that iron uptake and availability have some relation with the mechanism of strategy I in maize seedlings. For instance, under iron-deficient conditions, H_2_S could regulate the mRNA expression abundance of *ZmFRO1* and *ZmIRT1* in roots and leaves of maize seedlings. Taken together, these two strategies can increase iron uptake and availability, which will further promote chlorophyll content, chloroplast development, photosynthesis-related gene expression, and photosynthesis in maize seedlings.

In summary, results of this study further show that H_2_S is closely related to iron uptake, transport, and availability, consequently increasing chlorophyll biosynthesis, chloroplast development, and photosynthesis. Further studies are needed to understand and unveil the possible multiple pathways of H_2_S-regulated iron availability in plants. This report opens a new window for the study for H_2_S in plant nutrient availability, and adds fresh knowledge for the improvement of crop growth and production.

## Supplementary data

Supplementary data are available at *JXB* online.

Table S1. Sequences of forward and reverse primers used in qRT-PCR for gene expression analysis in maize seedlings.

Table S2. Procedures of dsDNA synthesis used in qRT-PCR for gene expression analysis in leaves of maize plants.

Table S3. TEM analysis of the effect of NaHS on the numbers of grana stacks and grana lamellae in mesophyll cell chloroplast ultrastructure of maize leaves.

Fig. S1. Effect of NaHS on biomass of iron-deficient maize plants during the period of growth.

Fig. S2. Time-dependent gene expression of *ZmYS1* in iron-deficient maize roots after NaHS treatment.

Supplementary Data
