# Supplementary material for: Hydrogen sulphide improves adaptation of Zea mays seedlings to iron deficiency
Source: J Exp Bot. 2015 Jul 23;66(21):6605–22. doi: 10.1093/jxb/erv368 (PMC4623679; doi:10.1093/jxb/erv368)
Supplement: Supplementary Data [file supp_66_21_6605__index.html]

Hydrogen sulphide improves adaptation of Zea mays seedlings to iron deficiency — Hydrogen sulphide improves adaptation of Zea mays seedlings to iron deficiency — Supplementary Data 

# Hydrogen sulphide improves adaptation of *Zea mays* seedlings to iron deficiency

## Supplementary Data

Data files

- Supplementary Data - Supplementary Data
