# Supplementary material for: Hydrogen sulphide improves adaptation of Zea mays seedlings to iron deficiency
Source: J Exp Bot. 2015 Jul 23;66(21):6605–22. doi: 10.1093/jxb/erv368 (PMC4623679; doi:10.1093/jxb/erv368)

Title: Hydrogen sulfide improves adaptation of *Zea mays* seedlings to iron deficiency

Authors: Juan Chen [1,2], Fei-Hua Wu [2, 3], Yu-Ting Shang [1], Wen-Hua Wang [2], Wen-Jun Hu [2], Martin Simon [2], Xiang Liu [2] , Zhou-Ping Shangguan [1] and Hai-Lei Zheng [2,]*

Table S1 Sequences of forward and reverse primers used in qRT-PCR for gene expression analysis in maize seedlings.

Gene name	Full name	Forward primer sequence (5' to 3')	Reverse primer sequence (5' to 3')
ZmMTN	S-adenosylhomocysteine nucleosidase	ACATAACAAGCATCGGATCG	GGATGAGGATTAGGTCTACA
ZmNAS1	nicotianamine synthase 1	CGCTGCTTACAGGAGTCCTA	TATTATCAATGTTTAGCGAC
ZmNAS3	nicotianamine synthase 3	TGCGTACACCACATCTGTGC	AATCGTACATCGAATCGAG
ZmDMAS1	deoxymugineic acid synthase 1	AGCTTCGAGAAGTGCTAGTC	TTACGCACGGTGACGGGACG
ZmTOM1	transporter of MAs 1	GTGCCGTACTTAAATGTGAA	ACGGAAGAAAGGTCCCCTAG
ZmTOM2	transporter of MAs 2	TACCTATCGCGGCTGCTTTG	GCGGTCGTACGGCGCAAGAA
ZmTOM3	transporter of MAs 3	TGCGGACACTGACTTTCGTG	TTGTTTTAGGAACCTGGATA
ZmNRAMP1	natural resistance associated macrophage protein	TTGCTGGGGTACTAAACGAG	CCTTGTCACGACATCTAGGT
ZmMATE2	multi-drug and toxin efflux 2	AGTTTCATCTTCGGATCCTC	CCAATGTAAGTCCTGGTTGA
ZmFRO1	ferric-chelate reductase 1	TTCGCTCTTCCAACTCCAACTTCTGT	TTGATTACTTCTTCACCTGTAGCGTCT
ZmIBP	iron bing protein	AACGGCTGCCTCAGTGGAAGAGAT	CAAGCGATATGACGAGGTGATTCTGT
ZmIRT	iron-regulated transporter 1	CCACCGTGTCATCGCTCAGGTTCTTG	TAGACCCATGCCCTCGAACATTTGG
ZmYS1	yellow stripe 1	CACGGAAATGGAGAATGCGAGATAC	ACATAGGTTGAACCCAACATGACAA
ZmST1	sulfate transporter 1	TTGGCAGACAATACTGATAGGAGCG	ATTAGACTAGCTGAAGGTGGGTTGA
ZmATPS	ATP sulfurylase	CCCAGAATCAACTGTTGTTGCGATC	TCCCGTGGTCAGCATCATAGAGGT

ZmAPR	adenosine 5'-phosphosulfate reductase	GGCTCCGAAATCGCCATCGCCTTCAG	TGCCGTAGTGCTTCTCCACCTTGTCG
ZmOASTL1	*O*-acetyl-L-serine(thiol)lyase 1	TCACCCAGCTCATCGGCAACAC	ACGCTACAGCAGGGCTCCATAATCT
ZmOASTL2	*O*-acetyl-L-serine(thiol)lyase 2	TCCAACTCCGACGAGAACCGCCAC	CGACGCACCCATCGGTCACCTTGTT
ZmDES	Cys desulfhydrase	TGAGATCCTGAGCTATGCTATGGTC	GGAGGCTGCTTCTTTAGGCTTTGA
ZmRBCL	ribulose-1,5-bisphoshate carboxylase large subunit	ACCTATTTGAAGAGGGTTCTGTTAC	GATACCGTGAGGCGGACCTTGGAA
ZmRBCS	ribulose-1,5-bisphoshate carboxylase small subunit	GCAGTGCGTCAGCTTCATCGCCTACAA	TCGACAACATCAAGCAGACGCAGTG
ZmpsbA	D1 protein	TTTACATTGGATGGTTCGGTGTTTTGA	ATCTACTGGAGGAGCAGCGATGAAGG
ZmPEPC	phosphoenolpyruvate carboxylase	CCATCTGCTGGCTTCTGGAGTTTC	ACCAGGGAGAGCCCGAACGTGAAAA
Zmactin2	actin 2	TTGATTATGAACAGGAGCTGGAGACT	AGCCACCACTGAGGACAACATTACC

Table S2 Procedures of dsDNA synthesis used in qRT-PCR for gene expression analysis in leaves of maize plant.

Gene name	Procedures of dsDNA systhesis
ZmMTN	10 min at 95 °C and 40 cycles of 95 °C for 30 s, 46 °C for 30 s and 72 °C for 20 s
ZmNAS1	10 min at 95 °C and 40 cycles of 95 °C for 30 s, 49 °C for 30 s and 72 °C for 20 s
ZmNAS3	10 min at 95 °C and 40 cycles of 95 °C for 30 s, 51 °C for 30 s and 72 °C for 20 s
ZmDMAS1	10 min at 95 °C and 40 cycles of 95 °C for 30 s, 56 °C for 30 s and 72 °C for 20 s
ZmTOM1	10 min at 95 °C and 40 cycles of 95 °C for 30 s, 61 °C for 30 s and 72 °C for 20 s
ZmTOM2	10 min at 95 °C and 40 cycles of 95 °C for 30 s, 53 °C for 30 s and 72 °C for 25 s
ZmTOM3	10 min at 95 °C and 40 cycles of 95 °C for 30 s, 52 °C for 30 s and 72 °C for 24 s
ZmNRAMP1	10 min at 95 °C and 40 cycles of 95 °C for 30 s, 51 °C for 30 s and 72 °C for 20 s
ZmMATE2	10 min at 95 °C and 40 cycles of 95 °C for 30 s, 59 °C for 30 s and 72 °C for 20 s
ZmFRO1	10 min at 95 °C and 40 cycles of 95 °C for 30 s, 52 °C for 30 s and 72 °C for 20 s
ZmIBP	10 min at 95 °C and 40 cycles of 95 °C for 30 s, 57 °C for 30 s and 72 °C for 20 s
ZmIRT	10 min at 95 °C and 40 cycles of 95 °C for 30 s, 54 °C for 30 s and 72 °C for 20 s
ZmYS1	10 min at 95 °C and 40 cycles of 95 °C for 30 s, 53 °C for 30 s and 72 °C for 20 s
ZmST1	10 min at 95 °C and 40 cycles of 95 °C for 30 s, 56 °C for 30 s and 72 °C for 20 s
ZmATPS	10 min at 95 °C and 40 cycles of 95 °C for 30 s, 55 °C for 30 s and 72 °C for 20 s
ZmAPR	10 min at 95 °C and 40 cycles of 95 °C for 30 s, 56 °C for 30 s and 72 °C for 20 s
ZmOASTL1	10 min at 95 °C and 40 cycles of 95 °C for 30 s, 58 °C for 30 s and 72 °C for 20 s
ZmOASTL2	10 min at 95 °C and 40 cycles of 95 °C for 30 s, 50 °C for 30 s and 72 °C for 20 s
ZmDES	10 min at 95 °C and 40 cycles of 95 °C for 30 s, 54 °C for 30 s and 72 °C for 20 s
ZmRBCL	10 min at 95 °C and 40 cycles of 95 °C for 30 s, 60 °C for 30 s and 72 °C for 20 s
ZmRBCS	10 min at 95 °C and 40 cycles of 95 °C for 30 s, 55 °C for 30 s and 72 °C for 25 s
ZmpsbA	10 min at 95 °C and 40 cycles of 95 °C for 30 s, 50 °C for 30 s and 72 °C for 24 s
ZmPEPC	10 min at 95 °C and 40 cycles of 95 °C for 30 s, 57 °C for 30 s and 72 °C for 20 s
Zmactin2	10 min at 95 °C and 40 cycles of 95 °C for 30 s, 55 °C for 30 s and 72 °C for 20 s

Table S3 Transmission electron microscopy analysis for the effect of NaHS on the numbers of grana stacks and grana lamellae in mesophyll cell chloroplast ultrastructure of maize leaves. Maize seedlings were pre-treated with 100 μM NaHS for 8 d and then grown in a nutrient solution containing 1 μM Fe(III)-EDTA or 50 μM Fe(III)-EDTA for 12 d. Data are presented as means ± SE. Different letters in line indicate significant differences at $P < 0.05$ from different treatments. –Fe+NaHS: seedlings were pre-treated with 100 μM NaHS and then treated with 1 μM Fe; +Fe: 50 μM Fe; +Fe+NaHS: seedlings were pre-treated with 100 μM NaHS and then treated with 50 μM Fe.

**Variables**	**-Fe**	**-Fe+NaHS**	**+Fe**	**+Fe+NaHS**
Grana stacks (number/chloroplast)	18.8 ± 2.1 a	23.9 ± 2.0 b	35.4 ± 5.6 c	51.9 ± 3.2 d
Grana lamellae (number/grana)	3.6 ± 1.2 a	6.5 ± 0.9 b	10.1 ± 2.1 c	19.2 ± 2.0 d

Figure S1 Effect of NaHS on biomass of iron-deficient maize plants during the period of growth. Maize seedlings were pre-treated with 100 μM NaHS for 8 d and then grown in a nutrient solution containing 1 μM Fe(III)-EDTA (–Fe) for 50 d. Data are presented as means ± SE.

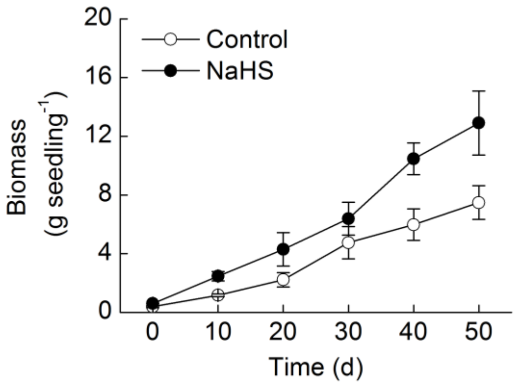

Figure S2 Time-dependent gene expression of *ZmYS1* in iron-deficiency maize roots after NaHS treatment. Maize seedlings were pre-treated with 100 μM NaHS for 8 d and then grown in a nutrient solution containing 1 μM Fe(III)-EDTA (–Fe) for 12 d. Data are presented as means ± SE.

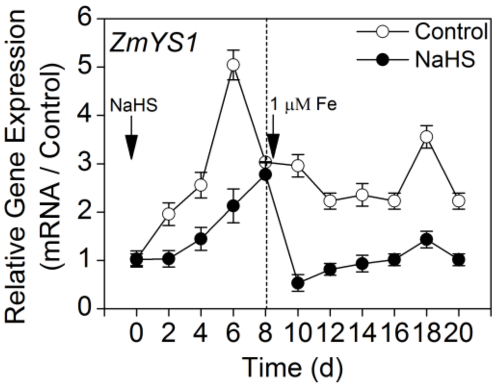

Supplement: Supplementary Data [file supp_erv368_jexbot150672_file001.pdf]
